# A solution to the enigma of the type locality of *Telmatobiushalli* Noble, 1938 (Anura, Telmatobiidae), a frog lost for 86 years

**DOI:** 10.3897/zookeys.1060.67904

**Published:** 2021-09-24

**Authors:** Claudio Correa

**Affiliations:** 1 Laboratorio de Sistemática y Conservación de Herpetozoos, Departamento de Zoología, Facultad de Ciencias Naturales y Oceanográficas, Universidad de Concepción, Barrio Universitario S/N, Concepción, Chile Universidad de Concepción Concepción Chile

**Keywords:** Hall’s water frog, International High Altitude Expedition to Chile, Loa River, Miño, northern Chile, Ollagüe

## Abstract

For 80 years, there were no sightings of the Andean frog, *Telmatobiushalli*, due to the ambiguity with which its type locality was described (“warm spring near Ollagüe”, northern Chile). The type specimens were collected during the International High Altitude Expedition to Chile (IHAEC) in 1935 and were subsequently described in 1938. In 2018 and 2020, two studies independently reported the rediscovery of the species, but they reached different conclusions about its identity and geographic distribution. In fact, the populations identified as *T.halli* in those studies are more phylogenetically related to other species than to each other, so they clearly do not belong to the same taxon. Although the study of 2020 is more in line with the geographic information of the description, it does not consider some bibliographic details and the transport limitations of the IHAEC. Here, based on a detailed analysis of the chronicles of the IHAEC and other bibliographic sources, I first refute the proposals of the 2018 and 2020 studies and then provide a possible solution. The combined information from the chronicles indicates that the type locality of *T.halli* is found at the sources of the Loa River, a different place from those identified in the two previous studies. By also incorporating geographic information of the time, I conclude that its true type locality is Miño, an abandoned mining camp located near the origin of the Loa River, where currently no populations of the genus have been described.

*Telmatobius* is one of the most representative and diversified genera of the highlands of the central Andes (Barrionuevo 2017). It currently comprises 63 species that are distributed in Ecuador, Perú, Bolivia, Chile and Argentina, mainly above 2000 m elevation (~ 1–30°S; Frost 2021). The distribution of the genus in Chile is restricted to the high Andean area of the extreme northeast of the country, where there are nine species, seven of them endemic (Correa 2019). The richness of the genus in Chile reached a maximum of 10 species with the description of *T.chusmisensis* Formas, Cuevas & Nuñez, 2006 (Formas et al. 2006), but since that date a series of taxonomic and systematic studies have better defined its diversity, phylogenetic relationships and geographic distribution in the country (Sáez et al. 2014; Victoriano et al. 2015; Fibla et al. 2017, 2018; Cuevas et al. 2020). Two of these studies, Fibla et al. (2018) and Cuevas et al. (2020), independently claimed to have clarified the location of the type locality and the identity of *T.halli* Noble, 1938, a species that had not been sighted for 80 years despite the efforts to find it (Formas et al. 2003, 2005; IUCN 2015).

*Telmatobiushalli* was described by Noble (1938) based on five adult females, one sexually immature female, and six larvae collected by Frank Gregory Hall during the International High Altitude Expedition to Chile (IHAEC) in 1935. The difficulty in locating the species was due to the vagueness with which Noble (1938) described the type locality: “Warm spring near Ollague, Chile, 10,000 ft. altitude, June, 25, 1935” (the correct spelling of the town is Ollagüe, which is at an elevation of 3705 m, 12,155.5 feet, according to Cuevas et al. 2020). Several populations of *Telmatobius* have been discovered near Ollagüe in the 21^st^ century (Fig. 1). Populations located immediately to the north were described as new species, *T.philippii* Cuevas & Formas, 2002 (Cuevas and Formas 2002) and *T.fronteriensis* Benavides, Ortiz & Formas, 2002 (Benavides et al. 2002). Then Formas et al. (2003), based on a morphological reanalysis of some type specimens, showed that *T.halli* is a good species compared to these new taxa. On the other hand, populations on the salt flats of Carcote and Ascotán, located south of Ollagüe (Fig. 1), were initially named as *Telmatobius* sp. (e.g., Sáez et al. 2014), but were later identified as Telmatobiuscf.philippii (Lobos et al. 2018) or *T.halli*, in the case of the population of Aguas Calientes of the Carcote salt flat (Cuevas et al. 2020; see below).

**Figure 1.**
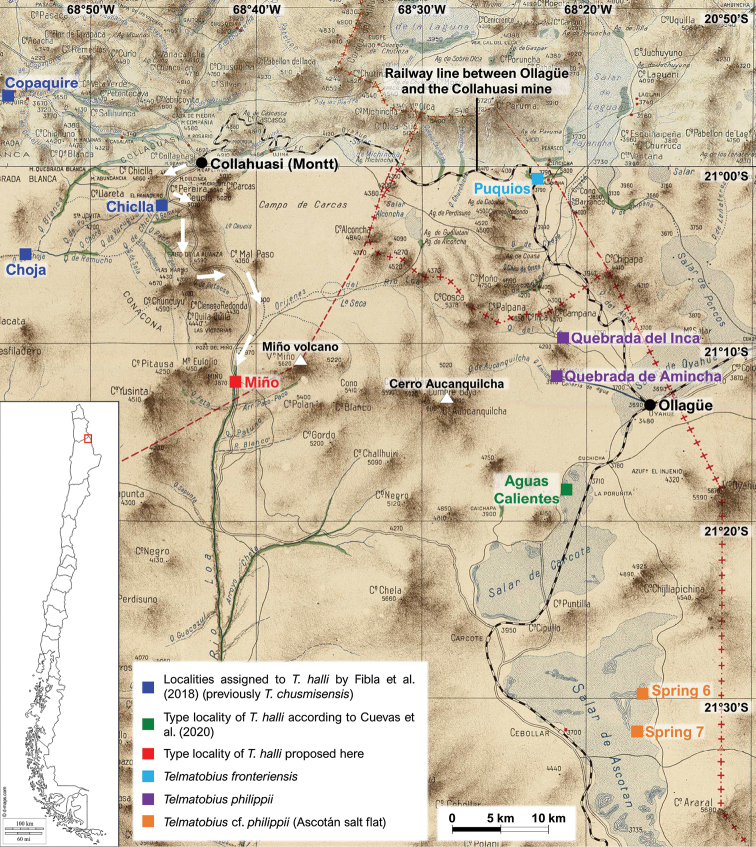
Upper area of the Loa River and surroundings, showing the location of populations recently assigned to *Telmatobiushalli*, its type locality according to this study and other *Telmatobius* populations near Ollagüe. The railway line that connected Ollagüe with the Collahuasi mine (to the Montt station) and the dirt road that connected the Collahuasi mine and Chiclla with Miño (white arrows) are indicated. Note that the road continues from Miño to the Carcote salt flat. The background map was constructed by joining two maps published by the Comisión Chilena de Límites in 1912.

Fibla et al. (2018) and Cuevas et al. (2020) used different approaches to solve the riddle of *T.halli*, although both reviewed key bibliographic sources of the IHAEC and compared the morphology of specimens of the type series with live individuals from candidate populations. Fibla et al. (2018) also performed a morphometric analysis of adults and molecular phylogenetic analyses, where all the species and some populations from north and south of Ollagüe were included. Two specimens from the type series were included in the morphometric analysis, but none was included in the phylogenetic analyses (probably due to the impossibility of obtaining DNA from them). Cuevas et al. (2020) redescribed the species using morphological characters of adults and larvae from their own candidate population, Aguas Calientes, which were compared with type specimens. Despite the soundness of their integrative approaches, Fibla et al. (2018) and Cuevas et al. (2020) arrived at different conclusions because they focused on candidate populations located in different zones (Fig. 1). In fact, the populations identified as *T.halli* in both studies are more related to other species than to each other (they even belong to different species groups) according to the most complete phylogenetic studies of Chilean species, in which type material of *T.halli* was not included (Sáez et al. 2014; Fibla et al. 2018). Consequently, there are currently two incompatible hypotheses about the identity and location of *T.halli*, which in both cases would be threatened by its very restricted distribution range.

The proposal of Cuevas et al. (2020) fits better with the little information given in the description (Noble 1938), since according to these authors *T.halli* inhabits the hot spring “Aguas Calientes” (21°17'44.4"S, 68°20'08.7"W, 3717 m), located in the northwest margin of the Carcote salt flat, 12 km southwest (straight line) of Ollagüe (Fig. 1). Although compelling, a careful examination of the chronicles of the IHAEC (Keys 1936a, 1936b; Keys et al. 1938; Dill 1979, 1980) shows that the solution of Cuevas et al. (2020) is highly unlikely and that it does not consider important background information contained in some of them. This information also allows me to rule out the proposal of Fibla et al. (2018) (see below).

Here I propose a solution to the enigma of the type locality of *T.halli* based on the descriptions of the activities of the IHAEC provided by two of its members, David Bruce Dill and Ancel Keys, historical maps and other bibliographic sources. To understand this new proposal, it is necessary to review in detail the information used by Fibla et al. (2018) and Cuevas et al. (2020).

Fibla et al. (2018) present the dates and altitudes of the places visited by the IHAEC (in their Table 1; see Keys 1936a, 1936b and Keys et al. 1938); then they summarize the itinerary of the expedition and mention a visit on a “free” Sunday to a “recreational area for the miners, located about 915 m a.s.l. lower than the mining area (at about 4000 m a.s.l.).” Referring to that visit, they transcribe a passage from Dill (1979), which describes the place and the circumstances in which Frank G. Hall collected the specimens that were used to describe the species. Considering these data, the dates of the expedition’s stay in the Collahuasi mine and the difficulty of movement at that time, Fibla et al. (2018) conclude that the populations of the Choja-Chijlla and Copaquire ravines, located near the mine, would correspond to *T.halli* (Fig. 1; throughout the text, Fibla et al. 2018 mention Choja-Chijlla together, but on their map they show them separately; here I located these two localities according to their map and I used the most common way of writing the second place, Chiclla). These populations were previously considered as *T.chusmisensis* in the molecular phylogenetic study of Sáez et al. (2014).

At this point, it is necessary to transcribe a more extensive fragment of the chronicle of the IHAEC of Dill (1979): “In 10 days [referring to the stay in Ollagüe], we were ready to move on to Montt [a train station very close to the Collahuasi mine] at 16,400 feet, the highest point reached by any standard gauge railroad. The rich underground copper mine was on standby at the time, manned only by a mine manager and a small maintenance crew. Within another 10-day period we had completed our observations and enjoyed another Sunday trip [Dill describes a previous one during the stay in Ollagüe], this time down 3,000 feet to a recreation area built for the mine staff. A concrete swimming pool filled with spring water was the major attraction. Greg [Frank Gregory Hall] searched the area for animal life and captured a frog that he preserved and eventually sent to the National Museum where it proved to be a new species. Appropriately it was named *Telmatobiushalli*. By this time it was late June and our train returned to Ollagüe.” Cuevas et al. (2020) provide a longer and modified version of this citation; I transcribed it literally, but inserted some clarifications in square brackets and added the last statement.

There are several elements in the preceding text that are important to understand fully the problem of the type locality of *T.halli*. First, the Sunday trip, an event described by Keys (1936b) and Dill (1979, 1980). This recreational trip, in which apparently all the members of the expedition participated, was made during their stay in Collahuasi (June 13 to 25) and should have taken place on June 23, as Fibla et al. (2018) inferred. Second, the altitude. Dill (1979) explicitly indicates that they descended 3000 feet (mentioned also by Dill 1980, see below), which means that on that trip they reached an altitude of 12,440 feet according to the height reported for Collahuasi (Montt) (4700 m, 15,440 feet; Keys 1936a, 1936b; Keys et al. 1938). Third, the concrete swimming pool filled with spring water. A very important detail is that Dill (1980) mentions that it was a “warm” spring (see below). And fourth, the train. The expedition moved from Chuquicamata to Ollagüe, and from there to Collahuasi (and from there back to Ollagüe), in four outfitted train cars, as Keys (1936a, 1936b) describe and show in photos.

Cuevas et al. (2020) also transcribe another text that mentions the same Sunday trip (Dill 1980): “The twelve days at Montt found us all busy except on Sunday when the resident engineers, Messrs. Packard and Bell, took us down about 3,000 ft to a concrete swimming pool fed by a warm spring, the origin of Rio Loa, where we enjoyed a swim. This had been a recreation spot for the mine staff. There was enough water running from the spring to support a flourishing green oasis. Hall, the naturalist, collected some specimens which he preserved and transported back to his laboratory. One specimen proved to be a new species of frog, which authorities at the National Museum appropriately named *Telmatobiushalli*. On June 25, our cars were pulled back to Ollagüe where they remained until our mission was completed in mid-July.” Again, Cuevas et al. (2020) provide a modified version of this citation, but I transcribed it literally and added the last statement. Here there are two additional details that are crucial to the argument that follows: the pool was at “the origin of Rio Loa” and the spring supported a “flourishing green oasis”.

Taking into account these elements, I will first discard the proposals of Fibla et al. (2018) and Cuevas et al. (2020) and then present a third account describing the finding of amphibians during the IHAEC that supports my own proposal.

The proposal of Fibla et al. (2018) rests strongly on the existence of known populations of *Telmatobius* near Collahuasi mine to the west, until then identified as *T.chusmisensis* (Sáez et al. 2014), and the difficulties of movement at the time of the IHAEC. Although this last argument is convincing, the coordinates that they provide (for Choja-Chiclla together and Copaquire separately; Fig. 1) are about 20–25 km from the mine, following the shortest route through the ravines, which can hardly be considered “near the camp” as the authors mention. In addition, they recognize that they did not find any pool with warm water like the one described in the chronicles, although they report the temperature of the water in Copaquire, which is warmer than that of another nearby stream, Chiclla. Nevertheless, the strongest argument to rule out the Fibla et al. (2018) hypothesis is that according to Dill (1980) (see quote above) the place where the pool was located is “the origin of Río Loa”. The same location had already been pinpointed by Keys (1936b) (“the source of the Rio Loa”), who also specified the altitude, 12,000 feet. Although the Choja-Chiclla and Copaquire ravines belong to the Loa River basin (Niemeyer and Cereceda 1984), they are formed by intermittent water systems that vanish into the plain where the Llamara salt flat is located, north of the Loa River. It is highly unlikely that the members of the expedition confused the streams located west of the mine with those that give rise to the Loa River, located south of the mine, considering that they had maps of the area (e.g., the ones shown in Keys 1936a and 1936b).

As mentioned above, the working hypothesis of Cuevas et al. (2020) is more congruent with the data associated with the description of *T.halli*, specifically the presence of a *Telmatobius* population in a warm (thermal as also they point out) spring near Ollagüe (Fig. 1). Additionally, the authors show a photograph of a pool that is roughly at the altitude of the site described by Dill (1979, 1980) and Keys (1936b) (see above), which could have been built on top of a previous one. This pool is surrounded by vegetation, which is consistent with the flourishing green oasis described by Dill (1980). Despite these similarities, the location of this site also does not correspond to the origin or source of the Loa River, as specified by Dill (1980) and Keys (1936b), since the Carcote salt flat is located in an endorheic basin that is not hydrographically connected with the tributaries of the Loa River (Niemeyer and Cereceda 1984).

There is another problem with the hypothesis of Cuevas et al. (2020): the logistics of the trip. The expedition arrived in Collahuasi from Ollagüe by train. Therefore, to travel to Ollagüe they would have had to take a train back (a journey of 91 km, Titus 1909) and then travel by another means of transport from Ollagüe to the Carcote salt flat (a journey of around 13 additional km, following the shortest route) (Fig. 1). Considering the conditions of that time, this would have been a fairly long journey in time and distance, without considering the costs and difficulties of moving a train (even assuming that they could move the train at any time, which is unlikely). In addition, the Sunday trip took place in the last days of their stay in Collahuasi, so it seems unlikely that they traveled to Ollagüe for a recreational trip, returned to Collahuasi and then almost immediately returned to Ollagüe (a detail that none of the chroniclers mention).

Although these last explanations respond to common sense, they are still only reasonable guesses about the movements of the expedition. Below I transcribe and translate literally a third account that is at the end of the section “Life in Collahuasi (4,700 meters)” from a chronicle of the IHAEC written in Spanish by Keys (1936a). This source, which was not consulted by Fibla et al. (2018), but is cited by Cuevas et al. (2020), clears up any doubts that could arise from my previous arguments: “Below Collahuasi, we examined the sources of the Loa River, the only one that reaches the sea in an area that spans 10° latitude. The water rises in a series of springs at a height of 3,700 meters, at the base of the Miño volcano (5,820 meters) and partly at the foot of Cerro Aucanquilcha. The fact that these sources are uniformly at the same level for a distance of several miles indicates the existence of horizontal stratification. Sedimentary formations of the Cretaceous surface a few hundred meters above the source line. Some of the springs are hot, but at this point the water is pure; some 50 kilometers below, salty springs begin to enter its waters, whose salt content is constantly increasing. We found many toads and tadpoles in the temperate ponds. The gorge of the Loa River begins a short distance from its sources; its roughness reminds us that the mountains and hills of this region owe their smoothness to the exceptionally small amount of water for erosion.” I think that this narration, which integrates several of the elements mentioned above (origin of the Loa River, an altitude of around 12,000 feet and springs of warm water), complements well the description of the Sunday trip. More importantly, it mentions the discovery of adult amphibians and larvae in that place. Although it does not explicitly mention that it was part of the Sunday trip, the concrete swimming pool or that G. Hall collected the amphibians, it is unlikely to be a different trip than the one described in the other two accounts transcribed above, since the activities of the IHAEC had another objective and there was probably not much time for recreation. In addition, part of the differences among the stories can be attributed to the fact that they were written by different members of the IHAEC.

A review of maps from the beginning of the 20^th^ century (Titus 1909; Oficina de Mensura de Tierras 1910; Comisión Chilena de Límites 1912; Richard Mayer 1917) and other sources allowed me to find a place called Miño, which is located at the source of the Loa River, at the base of the Miño volcano, and which has some characteristics compatible with the three narrations (Fig. 1). According to Riso Patrón (1924), (the settlement of) Miño is located at 3870 m (12,697 feet) and a road that connects Chiclla (near Collahuasi) with the Carcote and Ascotán salt flats passes through there (Fig. 1). Berenguer and Cáceres (2008) describe the ruins of this place, which they qualify as relatively large, indicating that it probably served as a post and mining camp from at least the 18^th^ century to the late 19^th^ century. There are currently at least three sets of ruins that can be clearly seen on Google Earth (satellite image of December 22, 2018; accessed on September 10, 2020), scattered around two waterways, in an area with abundant vegetation (21°11'49"S, 68°39'58"W, 3900 m, 12,795 feet). Although the satellite image do not show signs of the pool where the members of the IHAEC would have swum that Sunday, I propose that Miño is the true type locality of *T.halli*. There are no described populations of *Telmatobius* at this location, but about 180 km downstream, following the course of the Loa River, is the only known population of *T.dankoi* Formas, Northland, Capetillo, Nuñez, Cuevas & Brieva, 1999 (Formas et al. 1999).

A final comment. It is likely that we will never know why the type locality of *T.halli* was described so ambiguously and with an incorrect altitude (near Ollagüe at 10,000 feet), which has prevented this species from being found for 86 years. Considering this insurmountable limitation, it must be recognized that Fibla et al. (2018) and Cuevas et al. (2020) advanced in the right direction to solve the enigma, resorting to bibliographic sources, but paradoxically their conclusions imply that there are currently several populations of *Telmatobius* with the same name, which clearly do not correspond to the same species according to molecular phylogenetic studies (Sáez et al. 2014; Fibla et al. 2018). Therefore, the solution to this new conundrum requires intensive field work in and around Miño to try to locate the population originally described as *T.halli*.
